# Systematic design of health monitoring systems centered on older adults and ADLs

**DOI:** 10.1186/s12911-024-02432-3

**Published:** 2024-02-13

**Authors:** Francisco M. Garcia-Moreno, Maria Bermudez-Edo, José Manuel Pérez-Mármol, Jose Luis Garrido, María José Rodríguez-Fórtiz

**Affiliations:** 1 Department of Software Engineering, Computer Science School, University of Granada, C/ Periodista Daniel Saucedo Aranda, s/n, Granada, 18014 Spain; 2 Research Centre for Information and Communication Technologies (CITIC-UGR), University of Granada, Granada, Spain; 3 Department of Physiotherapy, Faculty of Health Sciences, University of Granada, Av. de la Ilustración, 60, 18016 Granada, Spain; 4 Instituto de Investigación Biosanitaria ibs.GRANADA, Granada, Spain

**Keywords:** Health monitoring, Activities of daily living, Conceptual models

## Abstract

**Background:**

Older adults face unique health challenges as they age, including physical and mental health issues and mood disorders. Negative emotions and social isolation significantly impact mental and physical health. To support older adults and address these challenges, healthcare professionals can use Information and Communication Technologies (ICTs) such as health monitoring systems with multiple sensors. These systems include digital biomarkers and data analytics that can streamline the diagnosis process and help older adults to maintain their independence and quality of life.

**Method:**

A design research methodology is followed to define a conceptual model as the main artifact and basis for the systematic design of successful systems centered on older adults monitoring within the health domain.

**Results:**

The results include a conceptual model focused on older adults' Activities of Daily Living (ADLs) and Health Status, considering various health dimensions, including social, emotional, physical, and cognitive dimensions. We also provide a detailed instantiation of the model in real use cases to validate the usefulness and feasibility of the proposal. In particular, the model has been used to develop two health systems intended to measure the degree of the elders' frailty and dependence with biomarkers and machine learning.

**Conclusions:**

The defined conceptual model can be the basis to develop health monitoring systems with multiple sensors and intelligence based on data analytics. This model offers a holistic approach to caring for and supporting older adults as they age, considering ADLs and various health dimensions. We have performed an experimental and qualitative validation of the proposal in the field of study. The conceptual model has been instantiated in two specific case uses, showing the provided abstraction level and the feasibility of the proposal to build reusable, extensible and adaptable health systems. The proposal can evolve by exploiting other scenarios and contexts.

## Introduction

The World Health Organization (WHO) defines quality of life as an individual's perception of their position based on their culture, values, goals, expectations, and concerns [1]. For older adults, good quality of life means living independently and feeling good while performing Activities of Daily Living (ADLs), considering life's physical, psychological, and social aspects [2]. Therefore, collecting data about physical, social, and cognitive health is necessary to promote healthy aging and enable older adults (people over 65 years) to live independently for as long as possible [3]. In addition, these data can be used for the early detection of behavioral changes and to identify risk factors related to functional decline in order to prevent problems in aging. For example, monitoring the participation in ADLs can be critical in preventing reversible issues that healthcare professionals can address.

Depressive and anxiety symptoms are common in older adults and can negatively impact their quality of life, leading to social isolation and other health issues. The aging process, coupled with a sedentary lifestyle, can also contribute to mental, social, and physical problems due to physiological changes such as muscle weakness, decreased aerobic capacity, and motor impairments, all of which can reduce a person's ability to perform ADLs efficiently and independently. Conditions such as frailty and dependence are predicted by observing declines in ADLs performance [4].

The ADL concept encompasses a holistic perspective due to the various dimensions (cognitive, social, emotional, etc.) required for completing these activities. Another critical aspect of monitoring ADL performance is the ecological perspective, which refers to observing a person in their natural environment (i.e., outside a laboratory or clinical setting). ADL assessments are less disruptive to the daily lives of older adults, more time and cost-efficient, and use fewer resources from the healthcare system compared to traditional assessments.

There is currently a focus in both political and research circles on improving the quality of life for elderly individuals. However, many previous observational studies and experiments have only considered isolated aspects of health, and the literature on this topic is limited in scope. Emerging Internet of Things (IoT) contributes with solutions, such as multi-sensing, intelligent systems, use of mobile/wearable devices, platforms, and data analytics to collect data from sensors providing physiological information and digital biomarkers related to health (e.g., heart rate, skin temperature, movement) and devices, providing context information (e.g., location, atmospheric pressure). Previous IoT systems are designed to address specific aspects of health, but they still need to provide a comprehensive solution.

Conceptual modeling is an effective way to manage complexity, particularly in system analysis and design. These models provide formal or semi-formal representations of relevant aspects of physical and digital realities. However, there is a growing need to reevaluate conceptual modeling in light of changing and emerging requirements. One of these emerging requirements is the need for human-centered design, which involves understanding the needs of people and designing solutions that meet those needs [5]. As a result, some works have called for a reconceptualization of conceptual modeling to better address these changing requirements [6].

The objective of this paper is to propose a conceptual model centered on the ADL concept that considers the various dimensions (social, emotional, physical, and cognitive) required for completing these activities, as well as the health status of the elderly people. Previous models have focused only on technological aspects (sensors, architectures, procedures, etc.) or the monitoring and recognition of specific ADLs, primarily in indoor environments and only considering the physical dimension of health. Our model aims to provide a general approach to model ADLs, including the human-centered design that considers the needs of older adults and how the design can meet these needs. The conceptual model presented here aims to provide transparency and comprehensibility in this domain and serves as a basis for developing multi-sensing, intelligent health monitoring systems. To that end, we also provide two instantiation examples of the model: a use case to monitor in real-time the frailty of the elders while performing an instrumental ADL (IADL), and another use case to assess dependence. We described the biomarkers or sensors and the machine learning pipeline to classify elders into frail, pre-frail or non-frail, and dependent or independent. These instantiations allow us to show the provided abstraction level of the model and the feasibility of the proposal to build reusable, extensible, and adaptable health systems in the field of study.

The paper is organized as follows. Section "Background" presents a literature review focused on the aspects of elderly people's health monitoring and ADLs, as well as the modeling of related concepts. Section "Literature review" presents the method followed in our research. Section "Methods" shows the results, the proposed conceptual model and two instantiation examples. Finally, Section "Results" summarizes conclusions and introduces future research.

## Background

The term ADLs involves all activities performed by human beings during their lifespan. One of the most accepted classification for ADLs is based on their level of complexity. This classification organizes activities from the basic ADL (BADL) (e.g. self-care activities, functional mobility, and the care of personal devices), through the instrumental ADL (IADL) (e.g. use of the phone, shopping tasks, and use of transportation), and up to the advanced ADL (AADL) (e.g. planning travels, and participation in events and meetings). Each ADL requires different body functions and structures from different dimensions (physical, cognitive, social, and emotional). IADLs require cognitive and motor complexity (executive functions), and imply an interaction with the social environment that surrounds the persons [7]. AADLs are the most complex ADLs as they involve voluntary physical and social functions, but are not essential to maintain independence. The performance of IADLs is an important health indicator for predicting mild or severe cognitive impairments, such as dementia in older adults [8].

Health systems can evaluate the health status by observing people's movement and exercise intensity, recognizing indoor and outdoor ADLs, and even detecting food intake, interactions with objects, relatives, and friends, etc. The identification of risks and anomalies is important to help the elderly and caregivers prevent dangerous situations [3]. Health systems based on intelligent systems aim to reduce hospital demands and costs. They include sensors that allow the continuous monitoring of different aspects of health, such as vital parameters (biomarkers), physical activities, and falls [9].

IoT includes sensors and devices with sensors, such as wearables and smartphones, used in the health environments to monitor biological, behavioral, and environmental data of people, because they are non-invasive, easily acceptable by subjects, and do not intrude users in their normal activities [10]. The most common sensors used in health as biomarkers are [9]: electrodermal activity (EDA), photoplethysmography (PPG), electrocardiography (ECG), electroencephalography (EEG), and skin temperature (SKT), among others. They collect physiological data such as heart rate, blood pressure, body temperature, respiratory rate, and blood oxygen saturation. Other physical reactions can also be measured with video or infrared cameras, microphones and electromyograms (EMG), for example, facial and body gestures. They can be combined with technologies to identify the dynamic position of people: Radio-Frequency Identification (RFID), Bluetooth Low Energy (BLE) or beacons, GPS, accelerometer (ACC) and gyroscope (GYR). Environmental sensors and sensors embedded in furniture, home appliances, walls and carpets also help to gather contextual information useful to know the user's behavior.

Once the sensors collect the data, it is necessary to analyze it to infer relevant information. Machine learning (ML) techniques are common in these situations because they provide better accuracy with big quantities of sensory data than other statistical analyses [11]. In particular, ML has been used to accurately recognize activities, detect health risk factors and specific health conditions such as frailty or dependence [12–14].

There are some important challenges in the development of the health monitoring systems: usability improvement, low-cost based solutions, data security guarantees, integration of devices, quality data collecting and processing, managing big data, and device power consumption [15]. Conceptual modeling could be useful for analyzing systems complexity and also to estimate the strategy behind the development of the software and the best devices to be used. Some of those challenges can be addressed by including them in the model.

## Literature review

The main problem with the heterogeneity of technologies in the IoT is that the observations are generated at different times, with different devices, protocols, vocabularies and data formats to be treated uniformly. Therefore, it is necessary to adopt generic solutions to describe and characterize systems, such as conceptual models that help to provide interoperability in IoT systems [16]. Several IoT initiatives try to model these environments. It is a field in continuous evolution and the reuse, evolution and extension of previous models is a common practice, and even some standardization bodies have proposed solutions.

One of the main initiatives is the Semantic Sensor Network model (SSN) by the World Wide Web Consortium (W3C), which developed a complete semantic model (based on all the sensor models in 2012) [17]. SSN describes the properties and measuring capabilities of sensors, taking also into account the processes or methods followed to measure (inputs and outputs if it is the case) and the stimulus observed, as well as their deployment in a system (platform and operation restrictions).

Other IoT models evolve by extending SSN, such as IoT-A (IoT-Architecture) or ARM (Architectural Reference Model) [17], which introduces the concepts of services, resources, and devices, and their interrelationships. These can be defined in a framework to enable the development of specific architectures with different levels of abstraction, independent of specific standards, technologies, implementations, or other low level details in systems development.

With the objective of processing more effectively IoT data, other initiatives move to the lightweight models, for example IoT-Lite [18], which also introduces the concepts of actuators and coverage; LiO-IoT (Light-weight Ontology-IoT) [19], that extends IoT-Lite adding Tag concepts and relationships; and IoT-Stream [20], which deals with data analytics. Even W3C updated its SSN with a core model less heavy called SOSA (Sensor, Observation, Sample, and Actuator) [21], which models: Sensor, Observation (made by sensors on observable properties by them), Sample (to connect an observation event with features of interest according to a strategy), and Actuation (by actuators or sensors with functionality).

Other models complement the sensor models with concepts needed to annotate the sensors and specifically the sensory data. For example, location models, such as Geo locate the sensors or data. Geo is composed of a few basic terms, such as latitude, longitude, and altitude. GeoSPARQL (Geo - SPARQL Protocol and Resource Description Framework (RDF) Query Language) is a standard for the representation and querying of geospatial data from the Open Geospatial Consortium (OGC) [22]. GeoJSON (Geo - JavaScript Object Notation) [23] is another example that describes geographic features, and geometric forms, such as Point, LineString, Polygon, MultiPoint, etc. Time ontology [24] represents topological (ordering) relations, duration, and temporal position (i.e., date–time information) with different time references (unix, geologic time, etc.).

All these models need taxonomies, which categorize the different devices, activities, etc. For example, the taxonomy of the Haddara & Howlader classification [25] describe specific characteristics of sensors: power (self-generated, integrated or external) configuration (portable devices or integrated in body or apparel) material (metals, conventional semiconductors, polymers, smart textiles or two-dimensional materials) sensing method (active or passive) sensing function (mechanical, electrical, optical, and chemical or chemical), etc.

The Quantity and Units (QU) model [26] focuses on quantities and units and supports different Systems Modeling Languages (SysML) users. For example, this model includes different dimensions to be measured such as *acceleration*, *luminousIntensity*, *temperature*, *frequency* and *velocityOrSpeed*. These dimensions could be considered as a full sample of observable properties in the way that SOSA considers them. Moreover, different properties are also considered in the QU model, for example, *conversionFactor* or *propertyType*.

Even some initiatives perform a step further and do not only create the taxonomy, but divide the taxonomy in levels. For example, Kamišalić et al. [27] present a wristband device taxonomy in three levels: the tracking raw input (sensory data), the raw output (sensors, devices and services), and the intelligent output (high-level events). The tracking raw input includes physiological human data such as heart rate, electrodermal activity, body temperature and body sugar. Human activity is also considered in this level, including proximity detection, motion and gestures. The last kind of data in this first level is the environmental data, which gathers data such as altitude, location, light, etc. The raw output considers three kinds of sources: sensors that show or share information, connected devices (e.g. phones, vehicles, etc) and connected services (e.g. weather, time, etc). The last level, the intelligent output, includes functionalities to recognize or evaluate different aspects related to health. Specifically, they are: stress and well-being, energy expenditure (calories), sleep quality, and recognition of activities, illness and distress.

Dealing with sensory data is usually noisy and faulty, so we also need to model the Quality of Information. The common quality concepts modeled are properties such as Completeness, Correctness, Concordance, Currency, Plausibility [28], Security [29], Access control in cloud data [30] and Confidence in the provenance of the data, PROV-O (Provenance Ontology) [31]. Another model [16] considers two dimensions of quality in which some of the mentioned properties also are included: generic data quality dimension (Accuracy, Confidence, Completeness, Data volume, Timeliness, Ease of access, Access security and Interpretability) and domain-specific data quality dimension (Duplicates and Availability). Even Zero model [32] uses blockchain to ensure two specific properties of quality, the Security and the Traceability of data.

IoT data normally gathers as streams of data, and annotating the atomic data within the streams has a high computational and time cost. Hence, some models annotate aggregated or processed data with analytics or machine learning algorithms. For example, SAO (Stream Annotation Ontology) [33], and IoT-Stream [20], allow knowing the different analytics followed in processing a stream and obtaining a final result, including pre-processing and classification or clustering models.

In the field of health, Health Level-7 (HL7) regulates the digital transfer of clinical and administrative data. It is a set of international standards and guidelines which provides a common vocabulary in order to interoperate between the endpoints. One of these standards is the Fast Healthcare Interoperability Resources (FHIR) [34], which divides the concepts into several levels. Level 1 provides the basic framework, mainly data types and formats. Level 2 deals with implementation and binding to external specifications, with concepts such as versions, databases, and security. Level 3 defines the patients and other concepts of healthcare systems such as devices, locations, etc. Level 4 annotates the records and processes, such as diagnosis, medication and financial issues. And level 5 provides the reasoning, and modeling concepts such as actuation plans.

The International Classification of Functioning, Disability and Health (ICF) [35] is one of the most used taxonomies to model Human Beings health-related domains classified from different perspectives: body functions and structure, individual and societal, environmental factors, activity and participation. This last domain includes the following classes: learning and applying knowledge, general tasks and demands, communication, mobility, self-care, domestic life, interpersonal interactions and relationships, major life areas, community, social and civic life. In a similar way, the World Health Organization (WHO) proposes different measures to assess health in three main dimensions: social, psychological and physical. The prevalence, incidence and severity of illnesses are the most commonly used rates to evaluate morbidity in a grading scale.

Several initiatives have already merged the concepts mentioned above [36]. For example, SmartEnv has applied them to older adults, extending SSN and representing different aspects of smart and sensorized environments [37]. These aspects are: observation/sensing, agents, activities/events, objects, network set-up, spatial and temporal aspects. The model annotates autonomous health systems used for elderly homes.

Regarding ADLs, regular activities performed in a house can be modeled with actions and concepts [38], for example, calling someone, etc, but it only models some activities and no instrumental activities. Other works model human activities in smart homes, emphasizing the time sequence, for example [39], specifying whether the activities are sequential or concurrent, and using rules to detect activities, for example, sleep takes place in bed and lasts 8 hours. However, these models only focus on BADLs performed in the house. In fact, almost all research focuses on identifying specific BADLs or detecting falls, contributing to the review of different kinds of sensors that could be used (and analyzing their limitations, such as cost, positions of wearable or environmental sensors, sampling frequency, and measurement range) [40], the creation of datasets following a protocol to gather data, or testing different techniques of analyses the data

In summary, many models cover different fields in great or shadow detail. However, we could not find any model covering all the concepts we need in a monitoring health system based on ADLs. Hence, we take some of the concepts from the models described above, and we extend these models with the concepts we could not find in previous literature.

## Methods

This research work is framed in a design science research methodology [41] to define a conceptual model as the main artifact and basis for the systematic design and implementation of successful systems centered on older adults monitoring within the health domain.

The design science research methodology can assist in detecting affordances, defining general design principles and guidelines, and devising new models/methods/techniques for the development of systems. The main objective is to bridge the gap between design theory and practice. Thus, design research has been applied to specific domains, areas and disciplines to solve design problems, for example in education [42] and information systems [43]. Specifically, we will follow an application of this methodology to design information systems, in particular the Design Science Research in Information Systems (DSRIS) methodology [44]. This methodology consists of several phases: 1) Background and awareness of real-world problem to be addressed; 2) Suggestion of various informed approaches that can contribute to solve the problem; 3) Development of artifacts explored in the previous suggestion phase; 4) Evaluation of the artifacts with direct iteration on the previous development phase; and 5) Conclusion of the accomplished research work.

**Fig 1:**
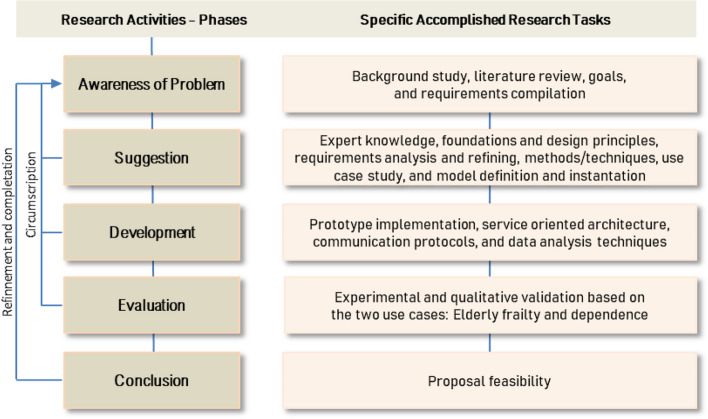
Methodology overview

Our main objective is the definition of a conceptual model as an artifact to provide knowledge and operational reusable support to the development of technology-based solutions in health considering human beings’ activities holistically and ecologically (ubiquitously). In Fig. 1 we show our research work according to this methodology.

In the *first phase*, we conducted a background study and a literature review by specifically examining up-to-date ADLs descriptions and evaluations. As in similar design processes and associated problems, we identified the need and feasibility of carrying out the ADL monitoring by using emerging technologies, but additionally, the evaluation of ADL performance in a holistic and ecological manner. Therefore, we derived the main goals and requirements to support processes.

In the *second phase*, expert knowledge was gathered by means of interviews, and theoretical foundations and design principles were identified by analyzing requirements. In addition, we studied candidate methods, techniques to analyze data based on machine learning, and technologies such as devices with built-in sensors. Small experiments and testing proofs were carried out on them to measure their capabilities. We also defined a conceptual model to characterize ADLs, health data, elderly people, data analysis and technologies to get data. The conceptual model serves as a common language for better communication between the involved experts (researchers and professionals) who work in different disciplines (health, engineers, data analysts, etc.).

In the *third phase*, we used the conceptual model to build a first prototype of the eHealth system to collect and analyze sensory data from these ADLs, so we planned to use a software structure based on service-oriented architecture, defining different kinds of services [13]. Services and microservices can be easily reused, extended, or coordinated to implement various use cases. Communication between services in IoT environments is often done using a publish/subscribe middleware. The services responsible for collecting sensory data must be able to communicate with devices and extract the data. Other services then implement a machine learning pipeline to process and analyze the raw data. This pipeline includes data collection and labeling, data preprocessing (such as segmentation, feature extraction, and missing value imputation), model creation (including training and testing with hyperparameter tuning and feature selection), and model performance evaluation using metrics such as accuracy and F1-score.

In the *fourth phase*, we show the results of the instantiation of the conceptual model elaborated in the second phase in two use cases: monitoring and analysis of frailty and dependence in older adults when performing the shopping ADL [13, 14, 41] ADLs. The two instantiations of the model, together with data collected from the development process (provided by the three developers involved in it) of the built prototype in the third phase (described in a previous paper [13]), allowed the experimental and qualitative validation of the proposal in the field of study. This informal validation is in terms of abstraction level provided by the conceptual model and the usefulness of the proposal, which includes making proper design decisions (e.g., the choice of the service-oriented architecture for software systems) to build reusable, extensible, and adaptable health systems centered on old adults. Finally, and as a direct consequence of the previous phase, in the *fifth phase* the positive results and first conclusions about the feasibility of the proposal will lead us to its application to other use cases.

Ethical considerations in the research were taken into account in the use cases, including the following aspects: ethical approval, voluntary participation, informed consent, anonymity and confidentiality of data, avoiding potential harm, and providing results to the participants who require them.

## Results

This section will be divided into two subsections, one for the model and another for the instantiation of the model and its validation.

### Proposed model

**Fig. 2:**
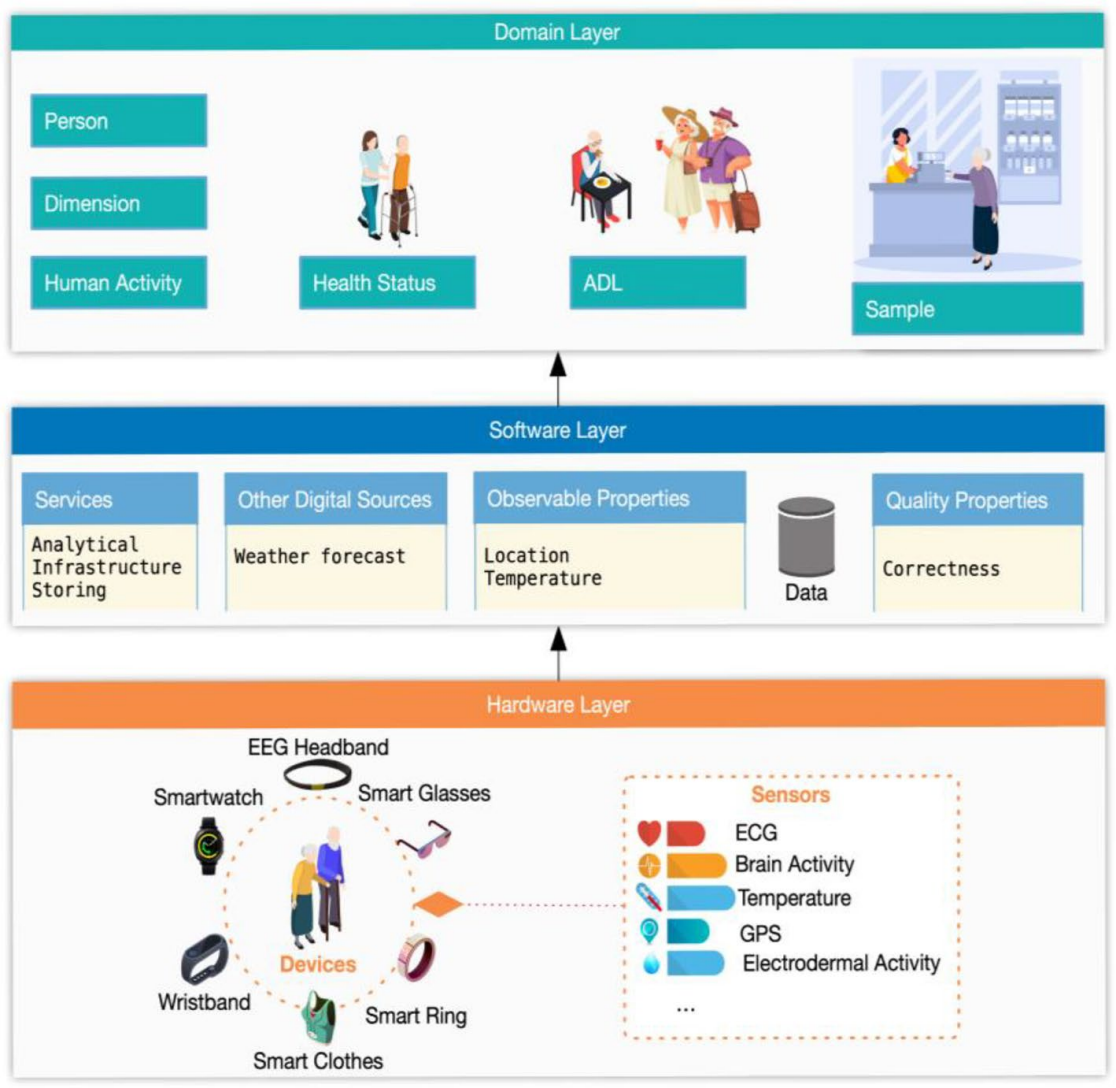
General overview of the conceptual model

**Fig. 3:**
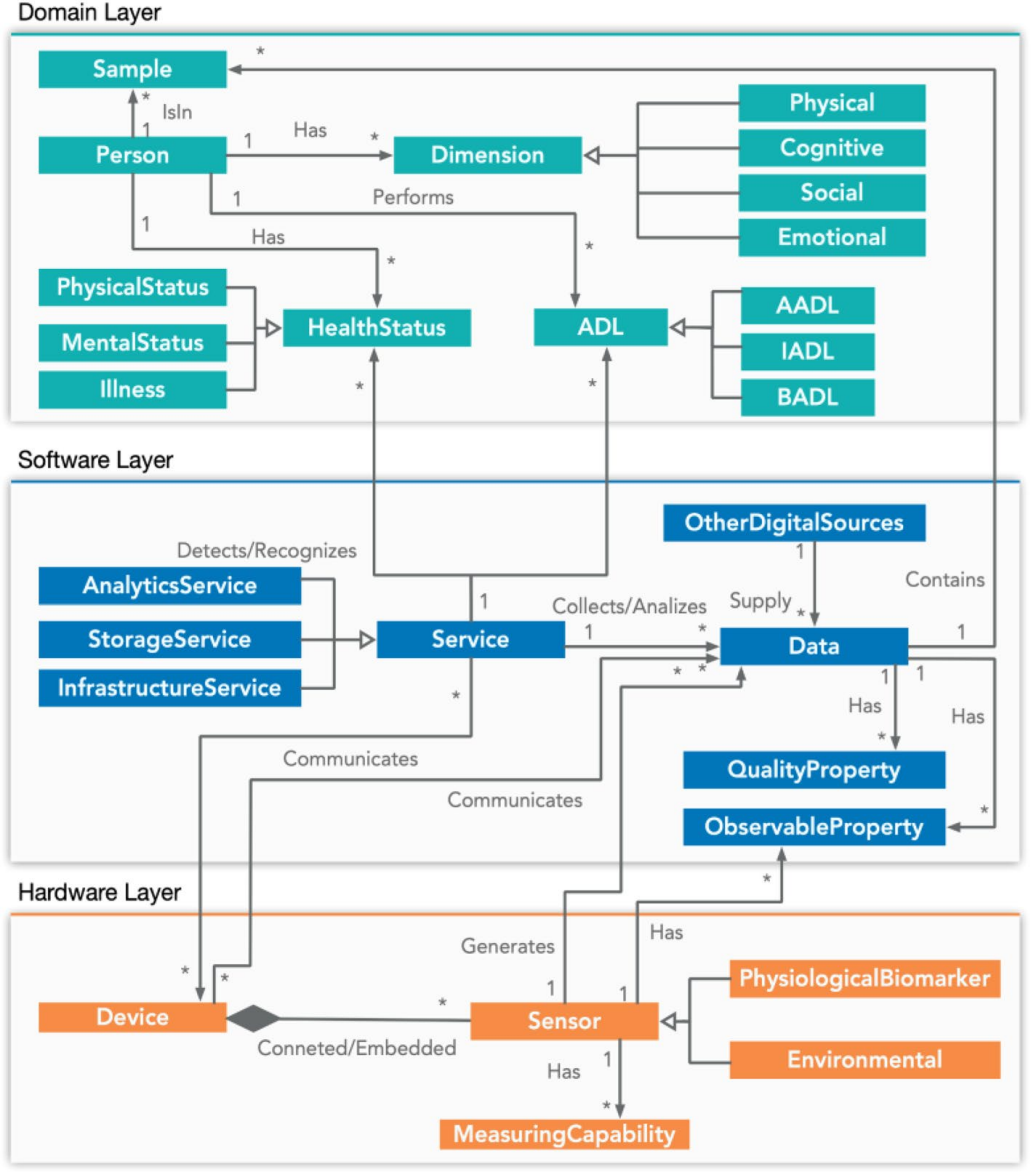
Conceptual model

We propose a holistic and ecological conceptual model for health focused on the monitoring of ADLs performance with sensors and data analytics. This model extends and reuses the models presented in section "Literature review", borrowing some concepts and relationships. Figure 2 shows the general overview of the model, and Fig. 3 shows the important concepts and relationships. Our model has three layers: hardware, software, and domain.

We use UML class diagrams (see Fig. 3) to depict the information structure, focusing on concepts and relationships. Attributes are detailed in tables (see Tables 1, 2 and 3). To instantiate and reuse our model, developers simply provide attribute values through class constructors. Additional responsibilities can be assigned to model concepts via associated methods/functions as needed. While UML is not a language that can be directly interpreted as code like RDF, numerous tools exist to automatically convert UML into various programming languages. Moreover, UML enjoys widespread usage, and most developers are proficient in translating it into executable code. This facilitates a semi-automated approach to system implementation based on our conceptual model. In addition, while the UML diagrams provide the structural foundation, we recommend the use of standardized data interchange formats, such as XML or JSON, for data exchange between other systems. This ensures that data generated in one system can be easily consumed, processed, and reused in another. Therefore, our model's structured representation in UML, combined with the use of standardized data interchange formats, ensures a consistent and normalized way of transferring information. This normalization process reduces the chances of data anomalies and ensures smoother data integration across platforms

**Table 1:** Characteristics of core concepts of the hardware layer

Device	Sensor	Measuring capabilities
Name	Name	Coverage
OperatingSystem	Domain	Latency
NetworkConnectivity	Configuration	Accuracy
Screen	Power	Frequency
Battery	SensingFunction	Resolution
Memory	SensingMethod	Sensitivity
ComputingPower	Material	Selectivity
	ObservedProperty	Precision
	StimulusDetected	ResponseTime
	Type	DetectionLimit
		inCondition

The *hardware layer* includes devices, defined as pieces of mechanical or electronic equipment that includes sensors. Examples of devices are: smartwatch, smart headband (e.g., wearables, mobile phones). Sensors are instruments that detect or measure a physical property and record, indicate or otherwise respond to it. Examples of sensors are: accelerometers or thermometers. A device can have several sensors embedded or receive data from external sensors. We have represented only the sensors most used in health: physiological or biomarkers and environmental sensors. Sensors have Measuring capabilities, it is properties associated with a measurement. Examples: accuracy, frequency. Table 1 shows one column for each concept: devices, sensors, and measuring capabilities.

The *software layer* (Table 2) includes services, categorization of data and specific properties. A service is a piece of software to manage the storage and analysis of data, as well as the infrastructure of a system. Examples of services are: communication services, sampling service. Each service has a name, is from a type or uses a method or technique which defines its purpose (e.g. communication, data storing or analytics), has several parameters and can have defined different performance indexes used to measure its accuracy, precision, efficiency and reliability, for example. There are specific services to communicate data between devices and servers, in which the services are deployed. For communication services, the most common ones implement request/reply (point-to-point communication) or Publication/Subscription (many-to-many communication) paradigms. Other specific services are specialized in data storage, for instance, data could be stored in a non-SQL database (local or cloud). Other analytic services process the data which usually apply machine learning techniques to identify or classify health status. The results of the analysis are sent to the devices used by elders or caregivers. This layer also defines the data. A data is an observation made by a human or a sensor device captured as a data point over a time instant or as a subset of data points over a time interval. Data can be communicated by devices or supplied by other digital resources such as external services. Each data has a timestamp, type, value, rank, format, and several attributes like the quality information attributes mentioned in the previous section (accuracy, completeness, confidence, availability, etc.). There are two attributes of the data, which are specifically included in this layer: Observable property and Quality property. The first one is an observable quality (property, characteristic) of a sensor, for example, temperature. The second one describes some values for the quality of a data stream, such as completeness or correctness.

**Table 2:** Characteristics of core concepts of the software layer

Service	Data	Observable property	Quality property
Name	Timestamp	PropertyName	Completeness
Type /Method/ Technique	Type	Type	Correctness
Parameters []	Value	ConversionFactor	Concordance
Performance indexes []	Rank		Plausability
	Format		

The *domain layer* (Table 3) represents the human beings (Person), and the information on their health status and Activities of daily living (ADLs) performance. There are three types of ADLS: AADLs, IADLs and BADLs. Each ADL has a name, a description and is performed in a place or a space (indoor or outdoor). Besides, an ADL can be carried out at the same time as others or sequentially (time sequence) and has different aspects that can be measured to assess its performance (Motions, Gestures, BodyAcceleration, Proximity, Duration, Intensity, etc.). We differentiate between two types of physical and mental health status, with different values for each health factor. Besides, human beings can be observed from different dimensions, (physical, cognitive, social, and emotional). The sample concept is also included in this layer. A sample is intended to be representative of Data on which Observations may be made. A sample is a subset of data taken in a situation. Examples of samples are values of the sensors of a smartwatch collected while performing an ADL.

**Table 3:** Characteristics of core concepts of the domain layer

Person	Health status	ADL	Sample
Identificator	Factor	Name	Description
Age	Type	Type	
Sex	Rank	Description	
	Value	Place	
	Severity	Time sequence	
		Measurements []	

Table 4 shows the terms used in our model. We borrow the terms “Device” ,“Sensor” and “Measuring Capabilities” from SSN. We add a taxonomy of sensors, used in the Health domain: Physiological and Environmental.

**Table 4:** Origin of the terms of our model

	SSN / SOSA [17, 21]	IoT-Lite [18]	IoT- Stream and SAO [20, 33]	QU [26]	FOAF [45]	HLT and ICF [35]	Haddara & Howlader [25]
Device	x						
Sensor	x						
Measuring Capabilities	x						
Service		x					
Analytics			x				
Observable and Quality Properties	x			x			x
Person					x		
Health Status						x	

We borrowed the term “Service” from the model IoT-Lite, and added to this term three subclasses “AnalyticService”, “StorageService” and “InfrastructureService”. These terms follow the types of services present in a microservice architecture. The “iot-lite:service” contains also related fields such as endpoint, type, and description of the interface. We borrowed the term “Analytics” from the IoT-Stream model to annotate the algorithms used to process the data, with the “parameters” and “paramValues” to annotate the hyperparameters of each machine learning algorithm. The terms ObservableProperties and QualityProperties measured by devices and sensors and managed by services are borrowed from SSN, and the taxonomies of mentioned properties from SSN, QU and the Haddara & Howlader proposal. The term “Person” is borrowed from the well-known model FOAF [45]. Although many models contain the concept of “Activity,” we could not find a model that distinguished between basic, instrumental, and advanced activities. Hence we extend the model with these concepts. The “HealthStatus” is based on the HLT and ICF classifications, and “Sample” is borrowed from SOSA to categorize the experiments.

As Table 4 shows, the use of only one of the conceptual models is not enough to characterize all the concepts and terms necessary to define the health system that we desire, including the monitoring of ADLs.

In addition to the terms taken from other models, new subclassifications have been added to the terms Device (Types of health sensors: Physiological and Environmental), Service (Subclasses of service: Analytic, Storage and Infrastructure Services) and Activity (Types of activities: Basic, Instrumental and Advanced activities).

### Model instantiation

In order to show how the model works and to check its flexibility to describe health systems in the field, this section illustrates its instantiation with examples in two use cases: frailty status detection and dependence assessment. We will describe below how we have instantiated the model to classify the frailty and dependence of the elderly. Frailty increases the risk of falls and the cognitive decline and can result in death, but can be prevented and reversed with intervention programs if it is detected early. Dependence is related directly to the performance of ADLs and implies a decrease in physical activity and social relationships, therefore therapists also can plan intervention programs to improve the state of the elderly and offer support when it is detected.

Two studies were conducted, involving 78 elderly participants. The inclusion criteria were: (1) ages ranged from 65 to 90 years old; (2) non-severe cognitive decline (using a cut-off score of ≥ 24 points in the Mini-Mental State Examination test); (3) non-perceptual alterations, determined by medical diagnosis report. Thus, the participants were included with perceptual alterations corrected with a support device, such as a pair of glasses or hearing aid; (4) walking with/without help (cane or walker).

We recruited a total of 78 participants from community day centers located in Granada, Spain. The participants comprised 69 women and 9 men. The average age of participants was 75 years, with a standard deviation of 5.735 years. In our frailty analysis, the distribution was as follows: 12 participants (or 15.38%) were classified as "frail", 47 (or 60.26%) as "pre-frail", and 19 (or 24.36%) as "non-frail". In terms of dependency, 30 participants (or 38.46%) were identified as "dependent", while the remaining 48 (or 61.54%) were deemed "independent”. The details of this experiment are out of the scope of this paper, but can be seen in references [13] and [14].

In both use cases we monitored elders with biomarkers in the form of wearables (smartwatches and wristbands) while they performed an IADL, in this case, shopping. In particular, for the frailty use case, the elders wore a smartwatch, the Samsung Gear S3. In the second use case, dependence, the participants wore the wristband Empatica E4. The biomarkers of each device are shown in Table 5.

**Table 5:** Physiological biomarkers

Device Name	Physiological Biomarkers	Use Case
Samsung Gear S3 smartwatch	Heart rate	Frailty
Empatica E4 wristband	Accelerometer x-axis Accelerometer y-axis Accelerometer z-axis Heart rate Electrodermal activity Infrared Thermopile	Dependence

**Fig 4:**
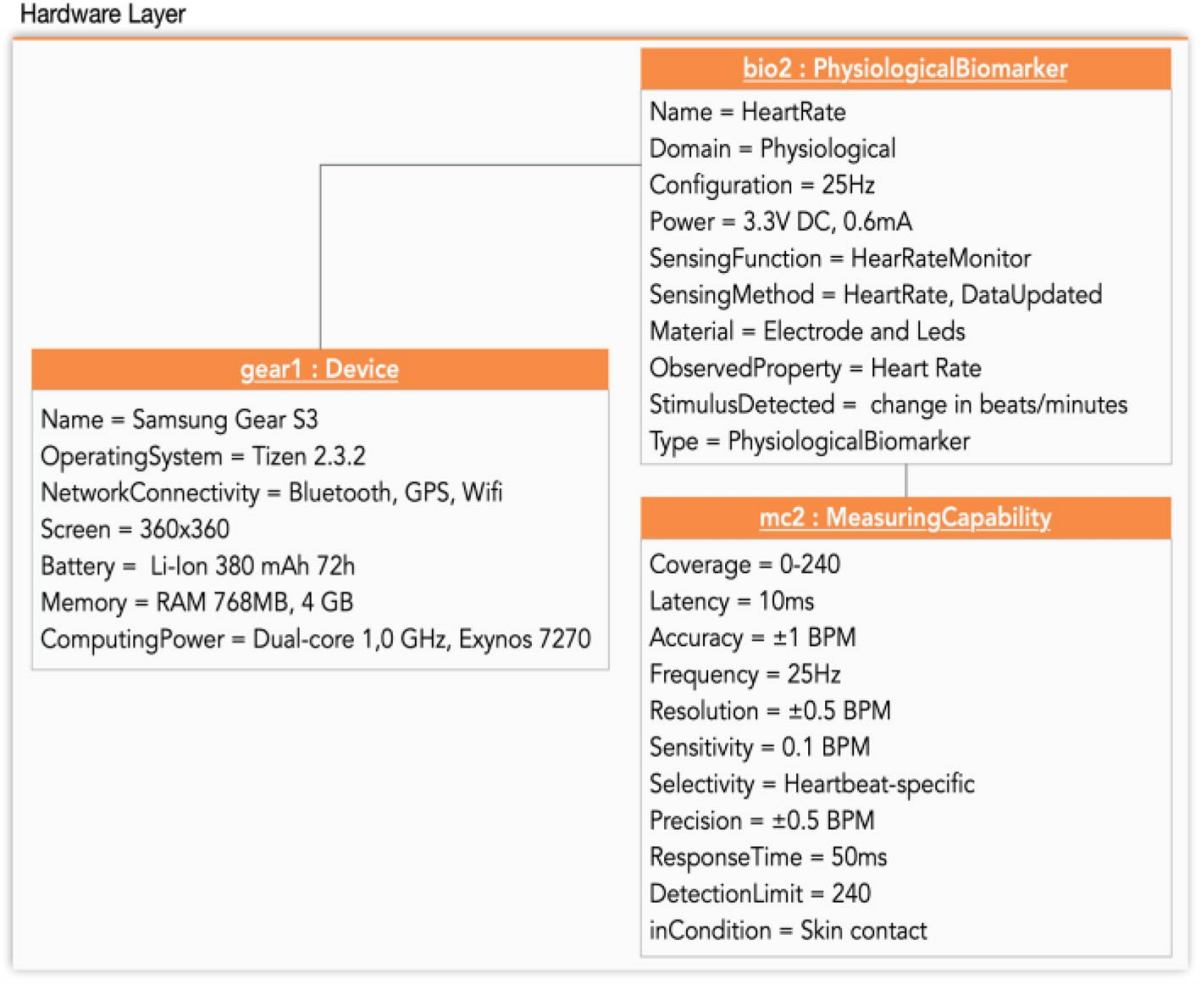
Hardware-layer Instantiation for the frailty use case

Figure 4. shows the instantiation of the conceptual model of the hardware layer for the frailty use case. The smartwatch has been instantiated (gear1: Device). The sensor of heart rate instantiated as (biomarker Heart Rate - bio2: PhysiologicalBiomarker) and its measuring capability (mc2: Measuring Capability).

**Fig 5:**
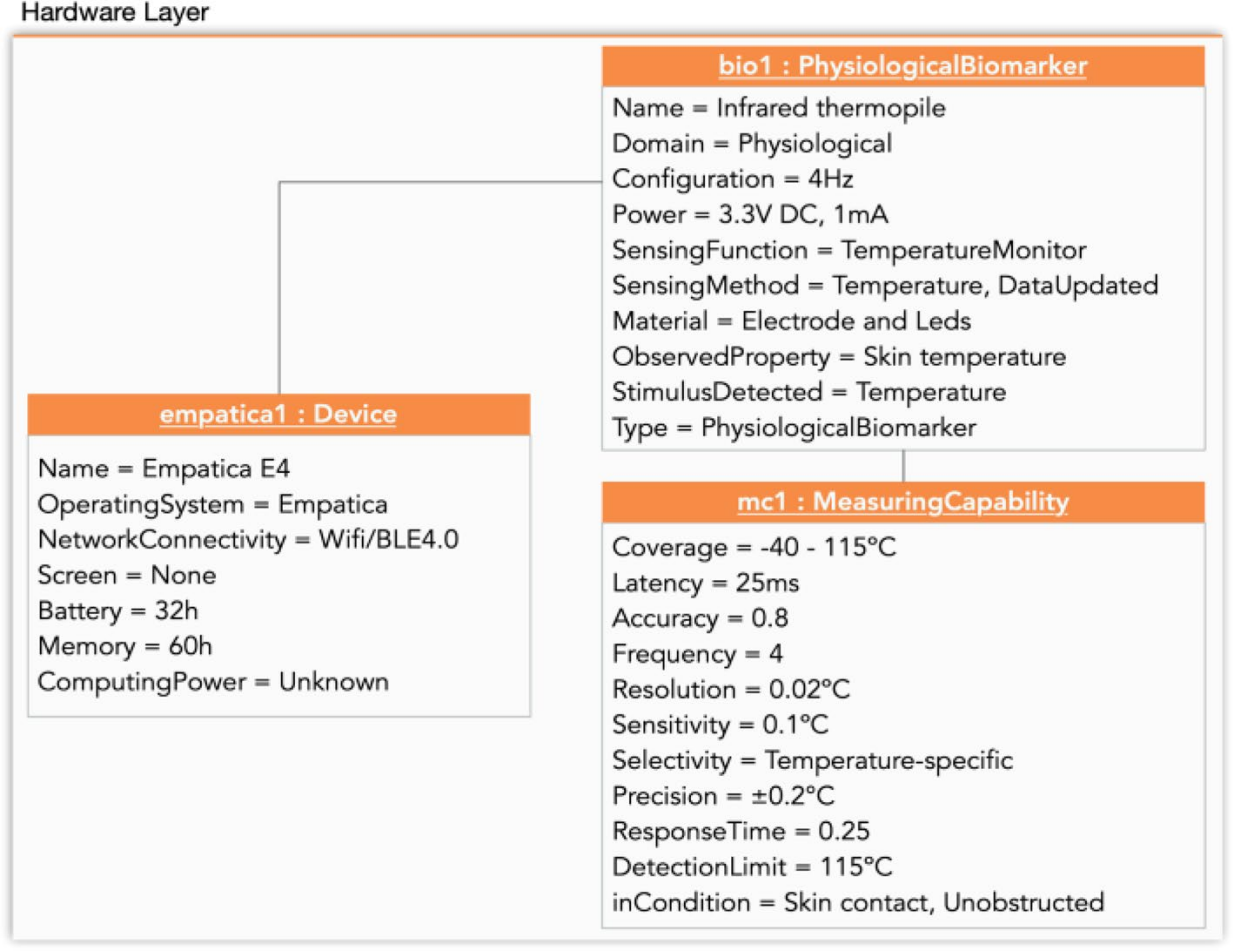
Hardware-layer Instantiation for the dependence use case (example)

In Fig. 5, we present the instantiation of the conceptual model of the hardware layer for the dependence use case. In this figure the wristband Empatica E4 has been instantiated (empatica1: Device). One of the sensors, temperature, has been instantiated (biomarker Infrared Thermopile - bio1: PhysiologicalBiomarker) and its measuring capability (mc1: Measuring Capability), is an example of how the other sensors could be instantiated.

For the first use case, we collected participants' frailty status based on the Fried classification for assessing frailty. With this test, we labeled all participants with their corresponding frailty status ("frail," "prefrail," and "non-frail"). In the second case, we use the Lawton and Brody scale for the assessment of dependence classifying the participants as “dependent” or “independent”.

**Fig 6:**
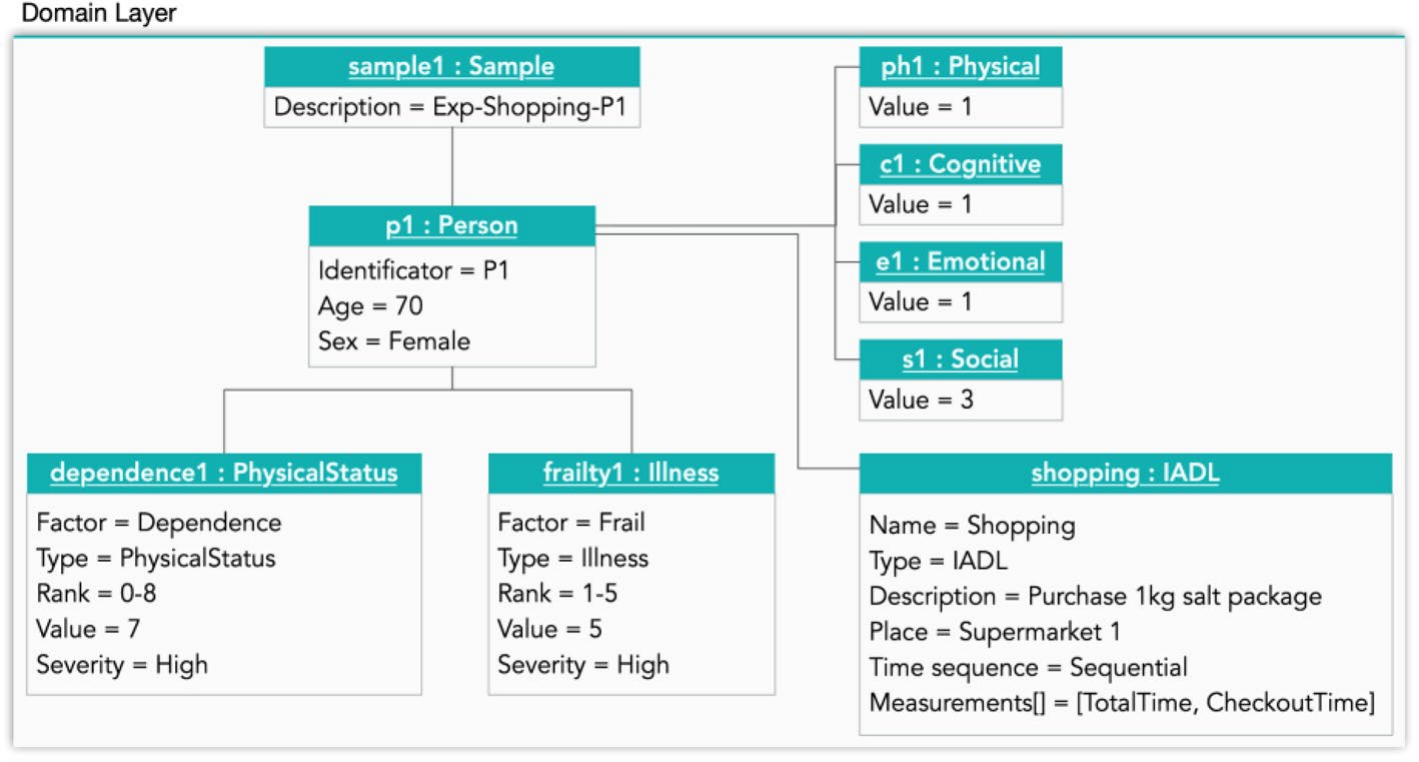
Domain-layer Instantiation for the Frailty and Dependence use cases

In both cases, the participants went to purchase a specific product at the nearest supermarket. Participants start in a chair without armrests and with the wearables in the non-dominant hand. Next, the participant gets up and goes to the supermarket, finds and picks up a 1 kg packet of salt, goes to the checkout, pays, returns to the starting point, and sits on the chair. In Fig. 6 the instantiation of the domain layer is shown for a sample of the shopping experiment in a supermarket (sample1: Sample), which assesses the dependence physical status (dependence1: PhysicalStatus) and a specific illness (frailty1: Illness) of one person (p1: Person), performing a specific IADL (shopping: IADL) and taking into account 4 multi-dimensional variables (ph1, c1, e1 and s1). The values of these variables come from results of tests carried out on the elderly to evaluate these dimensions of health, such as the Mini-Mental State Examination test, Fried test or Lawton and Brody scale. In our cases, the rank of the variables goes from 1 (worst value) to 4 (best value).

**Fig 7:**
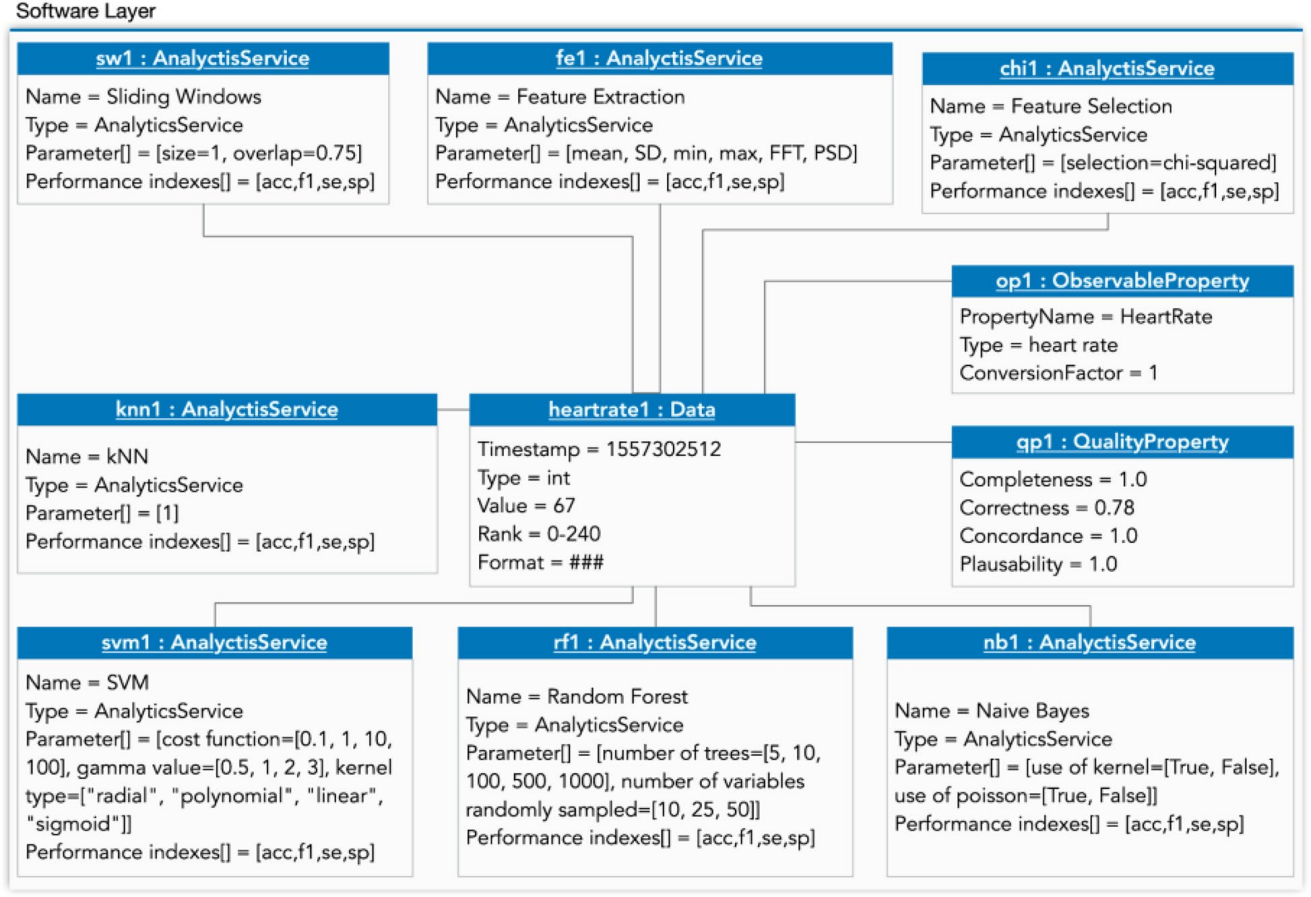
Software-layer Instantiation for the frailty use case

**Fig 8:**
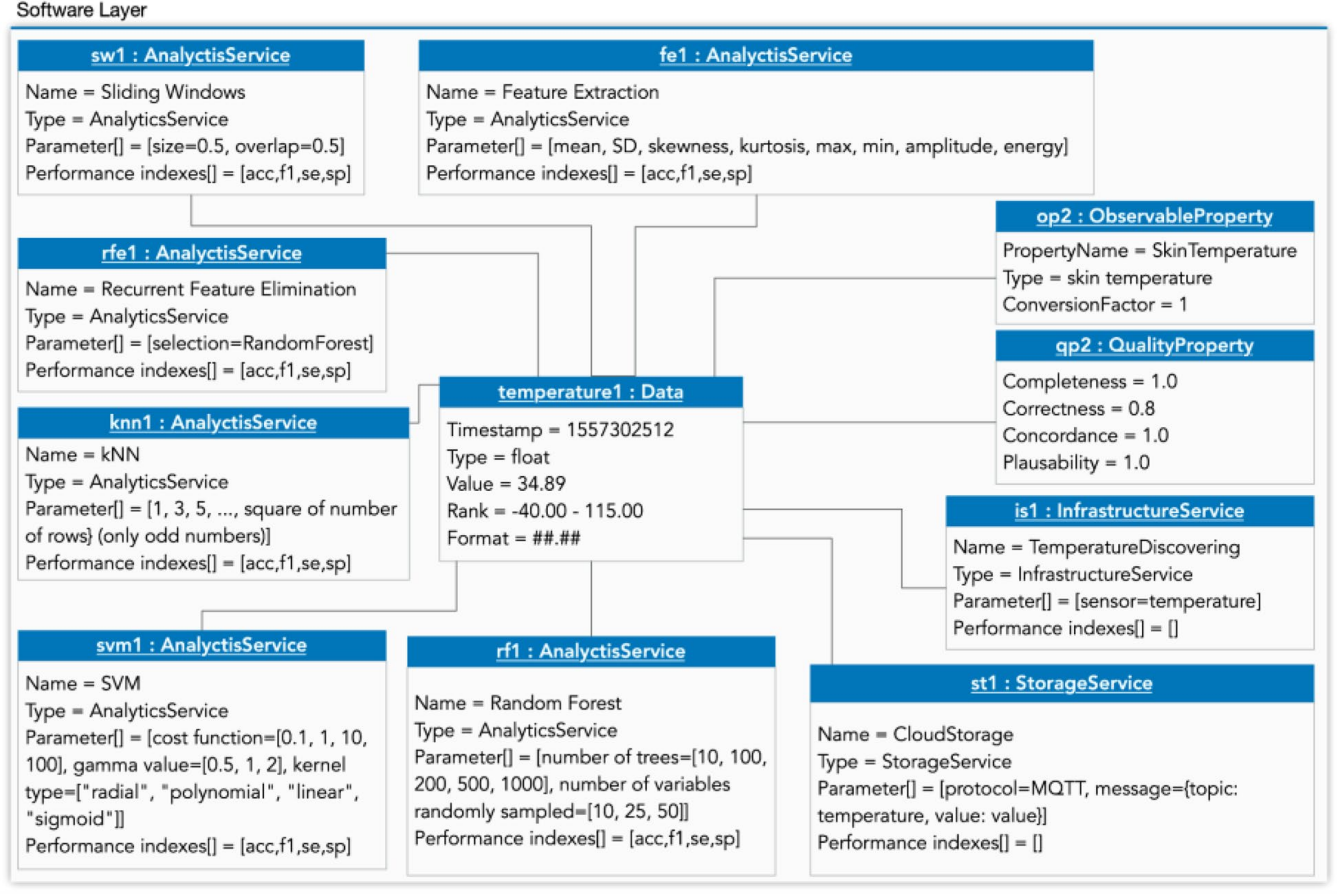
Software-layer Instantiation for the dependence use case

Figures 7 and 8 present the instantiations of the conceptual model of the software layer. Figure 7 shows the frailty use case, where the instance of data is heartrate1, and, for the dependence use case: the data of temperature is used—temperature1: Data.

We performed two preprocessing techniques sequentially, a sliding window—sw1: AnalyticsService—(different configurations are possible using parameter[] attribute, see sw1 in Figs. 7 and 8) [46–48] and a feature extraction (f1: AnalyticsService), calculating the features for each window. In Fig. 7, the features are mean, standard deviation (SD), skewness, kurtosis, maximum, minimum, amplitude, and the signal's energy; and, in Fig. 8 are mean, SD, maximum, minimum, FFT and PSD. Afterward, we performed a feature selection with random forest (RF) and Recurrent Feature Elimination (RFE) [49]: in Fig. 7, rf1: AnalyticsService and chi-squared in frailty use case (chi1:AnalyticsService), but only rf1 in Fig. 8. To classify the frailty of the elders, we applied four machine learning techniques with the hyperparameters tuned as in the literature [50–52] shown in Fig. 7 as knn1:AnalyticsService, svm1:AnalyticsService, rf1:AnalyticsService and nb1:AnalyticsService.

During our preliminary analysis for the frailty study, we found that the nb1:AnalyticsService classifier did not yield satisfactory outcomes. As such, when addressing the dependence study, based on our prior experience and aiming for efficiency, we strategically chose not to incorporate the nb1 classifier. Therefore, the last AnalyticsService is not applied in the dependence case.

Then, we performed 5-fold cross-validation [53] and calculated the performance index. We used four well-known performance indexes in the health field: accuracy (acc in Fig. 7), F1-score (f1 in Figs. 7 and 8), sensitivity (se in Figs. 7 and 8), and specificity (sp in Figs. 7 and 8). In addition, we also included in the case of dependence, Fig. 8, a service for infrastructure (is1:InfrastructureService), for discovering the temperature sensor, and another service for storage (st1:StorageService), to store the data from the Empatica device sensors, e.g. temperature (using MQTT protocol and the topic “temperature” with the value of this sensor).

Our results after executing the mentioned services for the frailty case are shown in Table 6, which shows a good performance for two of the algorithms (instances: knn1 and svm1) with all the performance indexes above 90%, in the classification of frailty and biomarkers shown previously in Table 4. The results of the performance in the evaluation of dependence are shown in Table 7, and they are also above 90% to classify between dependence and independence.

**Table 6:** Performance of frailty classification algorithms

Service	Accuracy	F1-score	Sensitivity	Specificity
knn1: AnalyticsService	0.9917641	0.9837171	0.9764216	0.9947197
svm1: AnalyticsService	0.9670102	0.9364576	0.9108271	0.9779242
rf1: AnalyticsService	0.8461648	0.6960141	0.6244533	0.8733734
nb1: AnalyticsService	0.6621256	0.4960688	0.4353061	0.7659894

**Table 7:** Performance of dependence classification algorithms

Service	Accuracy	F1-score	Sensitivity	Specificity
knn1: AnalyticsService	0.9963303	0.9951802	1.0	0.9941279
svm1: AnalyticsService	0.9862637	0.981914	0.9785293	0.9910763
rf1: AnalyticsService	0.9661179	0.9542469	0.9286523	0.9895499

Based on the consistent performance metrics from our 5-fold stratified cross-validation and the specific metrics we employed, KNN with K=1 demonstrated particularly strong results in our experiments. The detailed experiments and results can be seen in [13, 14].

### Validation

The model includes concepts and categories with a level of abstraction that allows the instantiation of different use cases, as it has been shown in the previous section for frailty and dependence. The model is general enough to be independent of specific use cases in the problem domain. Its structure in three layers (domain, hardware and software) allows us to deal with concepts and their relationships in a structured way, easily understanding them, and facilitating the selection of those that we need in each case. For example, in Fig. 6, the concept illness from the conceptual model has been used to describe frailty, and PhysicalStatus has been used for dependence, but MentalStatus has not been instantiated. The model is inherently flexible enough, for example, in our use cases, the AnalyticService has been instantiated several times with different values. Thus, each health system to be designed and implemented can be modeled in more detail in advance for a better understanding.

Besides, the developers informed about the presence of several quality properties in the proposal thanks to the design decisions made in the software development of the two health systems, for example, the choice of a service-oriented architecture and the use of API-Gateway communication pattern for the interaction between services/microservices [13]. The proposal fosters the reusability of existing services/microservices, for instance when it is required to use the same machine learning algorithm in different use cases, and also extensibility of the software by adding and implementing new required services, for instance implementing a different machine learning algorithm that can provide better analysis results for a specific domain problem in the field. Finally, the model is independent enough of the technology allowing the adaptation of health systems which can require the use of heterogeneous technologies such as devices with built-in sensors, operating systems, and other characteristics, according to their natural evolution.

## Conclusions and discussion

The decay in the elderly, which can be delayed or reversed, affects not only the physical dimension of health but also the cognitive, emotional, and social dimensions. We could consider all these dimensions by monitoring the elderly during their ADLs. Additionally, this monitoring is holistic and ecological. Systems using sensors and data analytics can help in the automation of health assessment by monitoring the performance of ADLs.

Design research helps experts to identify and devise models, vision, and contextualization to better understand the needs for the proposal and development of advanced monitoring systems in the health domain by using emerging paradigms (Internet of Things, eHealth) and technologies (wearable/mobile devices, software architectures, data analysis techniques, etc.). In particular, the holistic and ecological monitoring of health in older adults is challenging and complex. It involves dealing with socio-cultural and economic issues when transforming and combining traditional methods, procedures, and processes into new ones based on emerging technological solutions. Design research can be used to find principles, cases/scenarios/contexts, conceptual models, design guidelines and decisions, and for the validation and evaluation techniques suitable for developing new technology-based systems.

The literature has paid little attention to this topic. To the best of our knowledge, none of the previous models provides all the concepts we need, and none centers on the IADLs performed by the elderly indoors and outdoors. However, a holistic and ecological evaluation system for monitoring older adults can be established by observing the elders in their natural contexts during the performance of the ADLs involved in their routines.

The proposed model extends the literature by completing a comprehensive model to monitor health by evaluating IADLs performance with their dimensions (physical, cognitive, etc.). The model provides a generic and integral solution—extensible for different use cases (frailty, dependency, ADL recognition, monitoring, etc.)—and supports ecologic and holistic health evaluations. Design research has helped us improve practice and refine theory through iterative design, development, and analysis. The Applied method relates processes of development with outcomes. In particular, it provides valuable knowledge starting from a conceptual model artifact and, consequently, the derived artifacts that serve to build each specific health system. The conceptual model presented in the current work covers all the relevant elements to develop this monitoring system. Our solution uses several concepts from the previous literature. We have added the missing parts and linked them together. Our model serves as a basis for developing ecological and holistic health monitoring systems that consider several dimensions of health.

Although we tried to be careful in our validation by designing a general model that later on, we have validated with two use cases at two different temporal points, with real end users, we are aware that this internal validation could add some bias. We acknowledge the value that external validation holds. While our current validation has been internal, our intent moving forward is to introduce our system for external validation, encouraging a wider range of experts from both research and developer communities to engage with, utilize, and refine our model.

Another limitation of our model is that, as any model, it lacks a complete generalization that could be used as it is for all use cases. In our case we have focused on the monitorization of elderly people while they perform ADLs. Moreover, by definition, a model is an abstract representation of a system. Therefore, a model does not intend to include all the elements that are part of the system, which could lead to a complex and unmanageable model, but only the most representative elements for the intended use. However, as any model, it could be extended as necessary to include concepts for future use cases.

Our conceptual model is intended to represent the system requirements and serve as a reference for the design of health monitoring systems in the active and healthy aging domain that external research teams and developers can adopt to streamline their project initiation. Starting a new project often requires an intensive phase of research to identify the architectural components and their relationships. Using our model can significantly reduce this groundwork. By instantiating our conceptual model, teams can be assured of a comprehensive and holistic approach that has undergone some validation. This not only saves valuable time but also ensures that potential pitfalls or oversights are minimized. Moreover, the advantage of using our model, as opposed to developing a new one from scratch, lies in the accumulated insights and expertise it embodies.

This proposal still requires exploiting other scenarios and contexts. In this way, the conceptual model could embrace more concepts of the health domain. For instance, the domain layer in the conceptual model can increase its complexity as we classify the ADLs according to multiple criteria [38], not only based on their complexity level (BADL, IADL, etc.). Two examples of other criteria could be: the location, according to a rich combination of kinds of locations (indoor/outdoor, own/neighbor/friend/familiar house, sport/cultural/social centers, shopping/garden areas, etc); and whether the ADL is performed in a group or individually, the size of the group (small, medium, large) and characteristics of their members (e.g., age, illness, etc.). On the one hand, all these criteria could influence the level of performance when carrying out the same ADL. For instance, managing money could differ depending on the location (e.g., home compared to the bank office or supermarket) and how it is performed, individually or in a group (e.g., older person accompanied by a son). On the other hand, including information related to other classification criteria can more precisely determine the dimensions (cognitive, social, etc.) involved in the ADL. For example, managing money with her son can embrace not only the cognitive but also the social dimension. Similarly, when the older adult makes a phone call, the performance level can be influenced by the location (home, park, noisy street, etc.). Identifying the singularities of these criteria can help contextualize the ADL accurately, hence, derive the specific requirements of each scenario.

The results encourage sustained collaboration with stakeholders to refine the approach further and go a step forward with evaluations for additional use cases. In the future, we want to validate the proposed model by implementing a monitoring system for several use cases with older adults.

e-Health systems help health professionals to automate the assessment of health status. Our novel monitoring system may serve as a form for the evaluation of multiple health aspects at the same time, reducing time and cost for the health system and professionals. Additionally, the behavior of older adults under evaluation is expected to be more accurate/precise about their actual health status due to the reduction of multiple potential biases happening during the evaluation of the person in a clinical setting. The instantiations have allowed us to validate that the model can be useful in specifying different use cases in certain detail, for instance aspects that intervene in the monitoring of ADLs and health. Thus, experimental and qualitative validation of the model in the field of study through the instantiations for two specific case uses shows the desired abstraction level and, together with the making of suitable decisions of design, the feasibility of the proposal to build reusable, extensible, and adaptable health systems. The proposal can evolve by exploiting other scenarios and contexts.

## Data Availability

The datasets used and/or analyzed during the current study are available from the corresponding author upon reasonable request.

## Abbreviations

ICT: Information and Communication Technologies
ADL: Activities of Daily Living
BADL: Basic Activities of Daily Living
IADL: Instrumental Activities of Daily Living
WHO: World Health Organization
IoT: Internet of Things
EDA: Electrodermal Activity
PPG: Photoplethysmography
ECG: Electrocardiography
EEG: Electroencephalography
SKT: Skin Temperature
EMG: Electromyograms
RFID: Radio-Frequency Identification
BLE: Bluetooth Low Energy
ACC: Accelerometer
GYR: Gyroscope
ML: Machine Learning
SSN: Semantic Sensor Network Model
W3C: World Wide Web Consortium
IoT-A: IoT-Architecture
ARM: Architectural Reference Model
LiO-IoT: Light-weight Ontology-IoT
SOSA: Sensor, Observation, Sample, and Actuator
RDF: Resource Description Framework
OGC: Open Geospatial Consortium
GeoJSON: Geo - JavaScript Object Notation
QU: Quantity and Units
SysML: Systems Modeling Languages
SAO: Stream Annotation Ontology
HL7: Health Level-7
FHIR: Fast Healthcare Interoperability Resources
ICF: The International Classification of Functioning
DSRIS: Science Research in Information Systems
UML: Unified Modeling Language
XML: Extensive Markup Language
JSON: JavaScript Object Notation
SQL: Structured Query Language
non-SQL: Non-Structured Query Language
SD: Standard Deviation
FFT: Fast Fourier Transform
RF: Random Forest
PSD: Power Spectral Density
RFE: Recurrent Feature Elimination
MQTT: Message Queuing Telemetry Transport
KNN: K-Nearest Neighbors
API: Application Programming Interface
